# Supplementary material for: Vitamin D Modified DSS-Induced Colitis in Mice via STING Signaling Pathway
Source: Biology (Basel). 2025 Jun 18;14(6):715. doi: 10.3390/biology14060715 (PMC12190092; doi:10.3390/biology14060715)
Supplement: Supplementary file 1 [file biology-14-00715-s001.zip › Supplementary Materials S1: 16s-report of gut microbiota/Groups/diff_analysis/UnivarTestKEGG/figure/boxplot/top10.pdf]

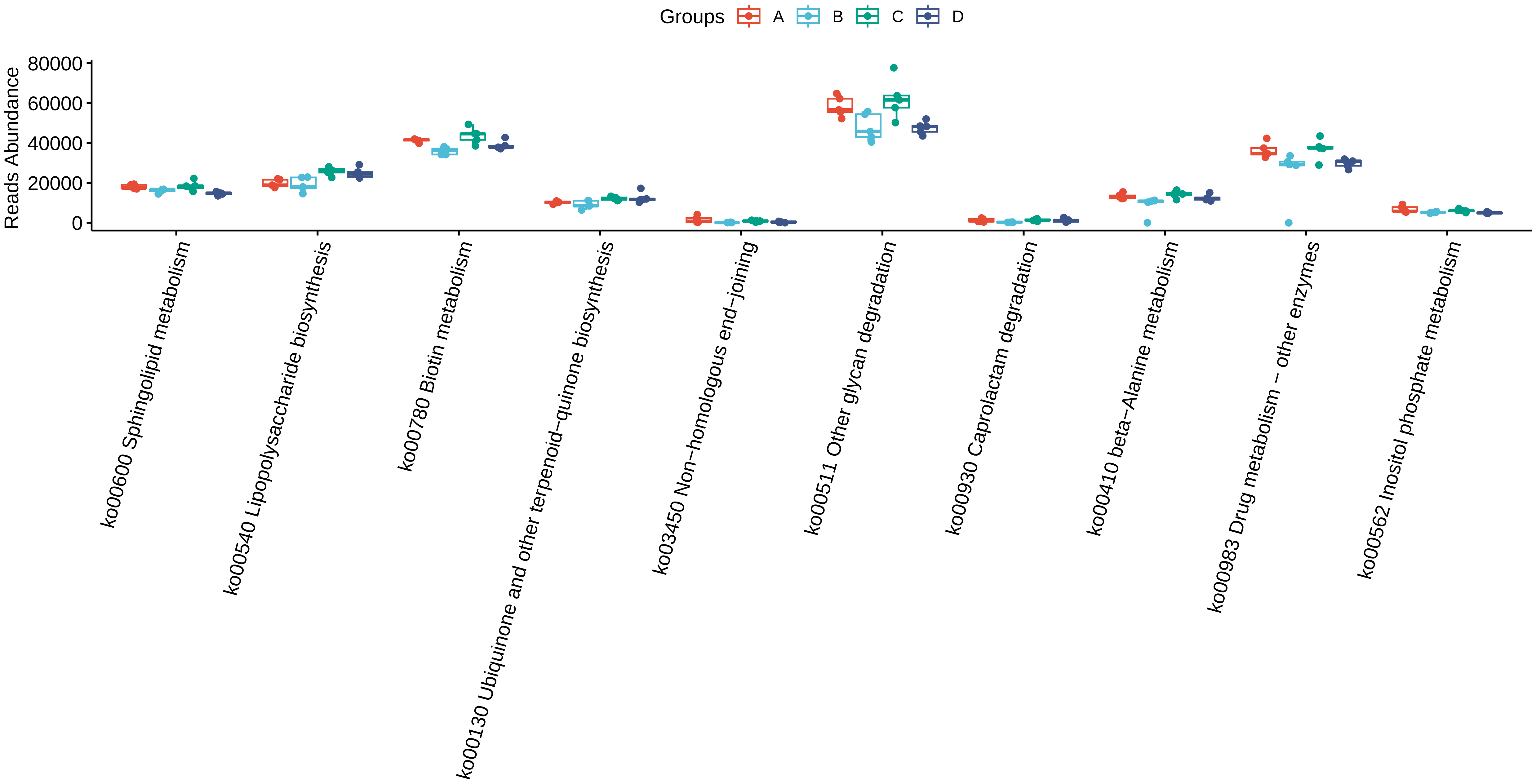
Groups
A
B
C
D
Reads Abundance
80000
60000
40000
20000
0
ko00600 Sphingolipid metabolism
ko00540 Lipopolysaccharide biosynthesis
ko00780 Biotin metabolism
ko00130 Ubiquinone and other terpenoid–quinone biosynthesis
ko03450 Non–homologous end–joining
ko00511 Other glycan degradation
ko00930 Caprolactam degradation
ko00410 beta–Alanine metabolism
ko00983 Drug metabolism – other enzymes
ko00562 Inositol phosphate metabolism
